# Promoter regions of *Plasmodium vivax *are poorly or not recognized by *Plasmodium falciparum*

**DOI:** 10.1186/1475-2875-6-20

**Published:** 2007-02-21

**Authors:** Mauro F Azevedo, Hernando A del Portillo

**Affiliations:** 1 Departamento de Parasitologia, Instituto de Ciências Biomédicas, Universidade de São Paulo, São Paulo, SP, Brasil. Avenida Lineu Prestes 1374, São Paulo, SP, 05508-900, Brasil; 2 Present address: Barcelona Center for International Health and Research (CRESIB), Rosselló 132, 4^a ^planta, 08036, Barcelona, Spain

## Abstract

**Background:**

Heterologous promoter analysis in *Plasmodium *has revealed the existence of conserved *cis *regulatory elements as promoters from different species can drive expression of reporter genes in heterologous transfection assays. Here, the functional characterization of different *Plasmodium vivax *promoters in *Plasmodium falciparum *using luciferase as the reporter gene is presented.

**Methods:**

Luciferase reporter plasmids harboring the upstream regions of the *msp1*, *dhfr*, and *vir3 *genes as well as the full-length intergenic regions of the *vir23/24 *and *ef-1α *genes of *P. vivax *were constructed and transiently transfected in *P. falciparum*.

**Results:**

Only the constructs with the full-length intergenic regions of the *vir23/24 *and *ef-1α *genes were recognized by the *P. falciparum *transcription machinery *albeit *to values approximately two orders of magnitude lower than those reported by *luc *plasmids harbouring promoter regions from *P. falciparum *and *Plasmodium berghei*. A bioinformatics approach allowed the identification of a motif (GCATAT) in the *ef-1α *intergenic region that is conserved in five *Plasmodium *species but is degenerate (GCANAN) in *P. vivax*. Mutations of this motif in the *P. berghei ef-1α *promoter region decreased reporter expression indicating it is active in gene expression in *Plasmodium*.

**Conclusion:**

Together, this data indicates that promoter regions of *P. vivax *are poorly or not recognized by the *P. falciparum *transcription machinery suggesting the existence of *P. vivax*-specific transcription regulatory elements.

## Background

Control of gene expression in malaria parasites seems unique among eukaryotes. Thus, global expression analysis of the intraerythrocytic cycle of *Plasmodium falciparum *at 1 h resolution demonstrated a tight regulation in which most genes are transcribed only once in the life cycle [1]. These include genes constitutively expressed in other organisms such as calmodulin, ribosomes, and histones, among others. Moreover, very few transcription factors mostly involved in RNA binding and possibly RNA stability have been annotated [2]. Furthermore, cooperation between introns and promoters has been demonstrated to be important for the silencing of *var *genes [3]. In addition, close to 10% of the parasite genes are transcribed by Pol II as antisense transcripts whose function, if any, is presently unknown [4,5]. Together, this data calls for a better understanding of control of gene expression in malaria parasites as it can reveal alternative control strategies.

The advent of transfection technology in *Plasmodium *allowed initiating functional studies of promoters [6-8]. Like higher eukaryotes, promoters of protein coding genes in malaria are transcribed by polymerase II and have a bipartite structure with a basal promoter followed by upstream regulatory elements [9]. Indeed, TATA boxes [10], INR elements [11] or downstream elements [12] could be acting depending on the promoter. Unlike higher eukaryotes however, the few *cis *acting elements functionally identified are distinct from their homologues in other higher eukaryotes [12-19], and the only transcription factor functionally characterized, the TATA binding protein (TBP), contains a C-terminus with low similarity compared to other TBPs [10]. In addition, the transcriptional machinery of malaria parasites is unable to recognize promiscuous viral promoters such as the CMV and SV40 promoters widely used in heterologous systems. Yet, important elements for transcriptional control are conserved among different *Plasmodium *species as promoters from different species drove the expression of reporter genes in heterologous transfection systems in the same pattern and activation timing of homologous systems [20-23]. To date however, no functional analysis of promoters from *P. vivax*, the most widely distributed human malaria parasites, have been conducted.

*P. vivax *infects reticulocytes, produces lower parasitaemia and rarely kills the host as compared to *P. falciparum*. Interestingly, its genome harbors regions with distinct AT-content in which central regions are GC-rich and syntenic with *P. falciparum *whereas subtelomeric regions are AT-rich and *P. vivax-*specific [24]. The objective of this study was to characterize *P. vivax *promoters having different AT-content through heterologous transient transfections in *P. falciparum*. We showed that *P. vivax *promoters are poorly or not recognized by the *P. falciparum *transcriptional machinery independent of the AT-content. Moreover, a functional regulatory motif identical in five *Plasmodium *species but degenerate in *P. vivax *was identified in the promoter region of the elongation factor one alpha (*ef-1α*) gene. This data suggests the existence of *P. vivax*-specific transcription regulatory elements.

## Methods

### Plasmid construction

Plasmid pE(A)b.luc.^D harbors the intergenic region between the two *Plasmodium berghei ef-1α *genes, the firefly luciferase gene and the *P. berghei dhfr *3' UTR and has been previously described [25]. Excepting for plasmid pPv-msp1, all other plasmids were constructed by digesting pE(A)b.luc.^D with *Nde*I and/or *Hind*III, making it blunt and replacing the *P. berghei ef-1α *intergenic region by the intergenic regions of the *P. vivax dhfr *(0.733 kbp), *msp1 *(1.3 kb), *vir3 *(1.8 kb), *vir24 *(1.3 kb), and *ef-1α *(1.4 kp). All intergenic regions from *P. vivax *were amplified with specific oligonucleotides (Table 1), subcloned into pGEM-T (Promega) or pCR4-TOPO (Invitrogen) and after being released from the vectors with appropriate restriction enzymes all inserts were cloned into pE(A)b.luc.^D. Plasmid pPv-msp1 was constructed by replacing the *hrp3 *promoter region from plasmid pHLH (kindly donated by Dr. Thomas Wellems) with 1,326 bp of the intergenic region from the *P. vivax msp1 *gene from the Sal-I strain. The *P. falciparum ef-1α *intergenic region was PCR amplified, cloned in both orientations in pPCR4-TOPO, digested with *Not*I, blunted, digested with *Spe*I and cloned in p0,5A(tetO)5' (*Nde*I/blunt/*Nhe*I), creating pPf-EF(A) and pPf-EF(B). p0,5A(tetO)5' is a plasmid derived from pE(A)b.luc.^D with approximately half of the *Pb ef-1α *promoter region. Plasmids with one or two copies of the EF motif were made by annealing the complementary oligonucleotides EFM01 and EFM01-2 (Table 1), cloning it in pPv-EF(B) (*Hind*III/blunt) to create plasmid pPv-EF(B)-HEF and then cloning it in pPv-EF(B)-HEF (*Nde*I/blunt) to create pPv-EF(B)-HNEF. Plasmids with mutated versions of the EF motif were made by site directed mutagenesis of pE(A)b.luc.^D. For all constructs, the thimidines (T) on positions 4 and 6 were replaced by cytosines (C). pE(A)b.luc.^D was hyper methilated, amplified by inverse PCR with oligonucletides mutPbEF1cc-F and mutPbEF1cc-R and transformed into DH5α-T1 cells (Invitrogen) to created pPb-1cc. Plasmid pPb-2cc containing the second EF motif mutated was created following the same methodology and using pE(A)b.luc.^D as a template and oligonucleotides mutPbEF2cc-F and mutPbEF2cc-R (Table 1). To make plasmid pPv-EF(B)-Pb0,2A, the *P. vivax ef-1α *intergenic region was cloned in pCR4-TOPO, digested with *Not*I, made blunt, digested with *Spe*I and cloned into pE(A)b.luc.^D (*Hind*III/blunt/*Spe*I). Plasmid pPv-EF(B) was created by digesting pE(A)b.luc.^D with *Nde*I, making it blunt and digesting it with *Kpn*I, releasing the *luc*-*dhfr *3' UTR cassette, which was cloned in pPv-EF(B)-Pb0,2A (*Spe*1/blunt/*Kpn*I). pPv-EF(B)-Pb0,2A contains a minimal promoter of *circa *0.2 kb from *P. berghei*. Authenticity of all plasmids was confirmed by DNA sequencing.

**Table 1:** List of oligonucleotides used in this study. Restriction sites or inserted mutations are represented in italics.

PvDHFR-F	5'GGGGTACCCTCGAGCAAGCGG3'
PvDHFR-R	5'TGCATGGGTTAAGCGGTTA3'
VIR3-F	5' GGTTTCATATAATTTTTAGA 3'
VIR3-R	5'CTTCTGATAATTACATGAGA3'
VIR23-24-F	5'*AAGCTT*GATGAAATTCAAGTTATGCT3'
VIR23-24-R	5'*CATATG*TGATAAGACATAGAAATTATATG3'
PvEF-F	5'TTTTGAATAATTTTTAAGTG3'
PvEF-R	5'TTTGAATAAGCTTTAATTTT3'
EFM01	5'TTTTTTTTGGGCATATATAAA3'
EFM01-2	5'AATATTTTATATATGCCCAAA3'
PfEF-F	5'TGTGTTTTTTCCTTACCCAA3'
PfEF-R	5'TTTGAATATATTTTTTTTAATTAATATAAG3'
mutPbEF1cc-F	5'TAAAAATATTATAAAATGCA*C*A*C*AATGTAGGG3'
mutPbEF1cc-R	5'TGCATTTTATAATATTTTTATTTATTTTAATA3'
mutPbEF2cc-F	5'TTATTTTTATACATATTTTTATATATTTTTTG3'
mutPbEF2cc-R	5'AAAAATATGTATAAAAATAA*G*T*G*TGCACTAAAT3'
FPv-msp1	5'GGGGTACCAGCACACCAAAGTGG3'
RPv-msp1	5'CCAATGCATGCATTTTCGAATTCGTCTA3'

### Parasite culture and transfection

The *P. falciparum *3D7 clone was continuously cultured *in vitro *[26] and transiently transfected as described elsewhere [27,28]. Briefly, 100 μg of each plasmid were used to electroporate 600 μl of uninfected red blood cells and this mix was added to about 10^7 ^parasites, which were kept in culture. Parasites were harvested 4 days after eletroporation and luciferase assays performed according to the manufacturer's instructions (Promega).

### Luciferase assay

Erythrocytes were harvested by saponin lysis, parasites washed twice in PBS and pellets ressuspended in 50 μl of 1× lysis buffer (Promega). To 20 μl of lysed parasites, 100 μl of luciferase essay reagent (Promega) were added and luciferase activity measured in the Lumat LB 9507 luminometer (EG and G Berthold) for 45 seconds. Reporter activity values represent the mean of at least three independent experiments done with two or three different DNA preparations. They are expressed in relation to the values of a reference control plasmid indicated in each experiment. Student's t test was used to determine the statistical significance of the data (p value ≤ 0.005).

### Bioinformatics analysis

Sequences of the intergenic region between the two *ef-1α *genes of six *Plasmodium *species (*Plasmodium knowlesi, Plasmodium reichenowi, Plasmodium yoelii, P. falciparum, P. berghei *and *P. vivax*) were retrieved from PlasmoDB [29]. The Gibbs matrices algorithm [30] of the Regulatory Sequence Analysis tools [31] was used to search conserved motifs in these intergenic regions. This algorithm finds optimized local alignments in related sequences in order to detect short conserved regions or motifs that may not be in the same positions. The matrix length was set from 4 to 20, which allowed the detection of very short and also longer and more complex conserved sequences. Matrices generated, which represent the nucleotide conservation in each position of the motifs, were used to search for the motifs positions, copy number and conservation using the Patser algorithm [32]. The Patser algorithm was applied to each of the matrices generated from the Gibbs analysis and sequences of the intergenic regions. The Alphabet parameter was set to a:t 0.4 c:g 0.1. Sequences of the most conserved motifs were input in the WebLogo program [33] to generate the visual representation of the consensus sequence, which were then compared to the sequence of the motif in the *P. vivax *intergenic region.

## Results

### Upstream regions of the *P. vivax dhfr*, *msp1*, and *vir3 *genes are not recognized by the *P. falciparum *transcription machinery

**Figure 1:**
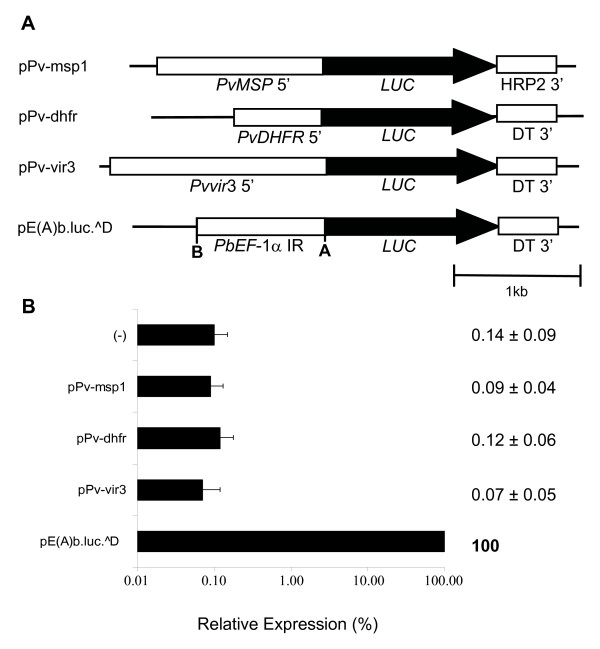
**Upstream regions of *P. vivax msp1*, *dhfr *and *vir3 *are not recognized by *P. falciparum***. A. Luciferase reporter plasmids constructed with *P. vivax *upstream regions. *PvMSP *5' – 5' upstream region of *P. vivax merozoite surface protein one; PvDHFR *5' – 5'upstream region of *P. vivax dihydrofolate reductase *gene; *Pvvir*3 5' – 5' upstream region of *P. vivax variant gene *3; *Pb ef-1α *IR – *P. berghei elongation factor one alpha *intergenic region; *LUC *– luciferase; DT3' – *P. berghei dhfr *3' UTR; HRP2 3' – *P. falciparum histidine rich protein *2 3' UTR. pE(A)b.luc.^D has the *P. berghei ef-1α *intergenic region. B. Transient transfection in *P. falciparum*. Plasmids were transiently transfected in *P. falciparum*, parasites kept in culture for four days and luciferase activity detected. Luciferase activity is expressed relative to pE(A)b.luc.^D used throughout this study as the positive control. Negative control (-) refers to luminescence measures of the substrate alone or plasmid with luciferase and malaria 3' UTR but no promoter. Log scale was used. Values represent the mean of at least three independent experiments done with two or three different DNA preparations. Bars represent standard deviations.

Initially, luciferase reporter plasmids containing 0.733 kB of the 5' upstream region of the *P. vivax dhfr-ts *gene with circa 49% AT-content (pPv-dhfr), 1.326 kbp of the upstream region of the *P. vivax msp*1 gene with *circa *59% AT-content (pPv-msp1), and 1.818 kbp of the upstream region of *vir*3 gene with *circa *81% AT-content (pPv-vir3), were constructed (Figure 1A). Surprisingly, transient transfections of these recombinant plasmids into *P. falciparum *produced luciferase activity values similar to the background where the luminescence of the substrate alone or of the plasmid p-.luc^D, which has luciferase, *P. berghei *3'UTR, but no promoter, was measured and used as negative controls. This contrasts with parasites transfected with plasmid pE(A)b.luc.^D which contains a *P. berghei *promoter and had been previously shown to give high luciferase values in transient transfections assays in *P. berghei *and *P. falciparum *[23,25]. This plasmid was used as reference and positive control plasmid throughout this study (Figure 1B).

### Entire intergenic regions of *vir *and *ef-1α *genes from *P. vivax *contain minimal promoter elements poorly recognized by the *P. falciparum *transcriptional machinery

**Figure 2:**
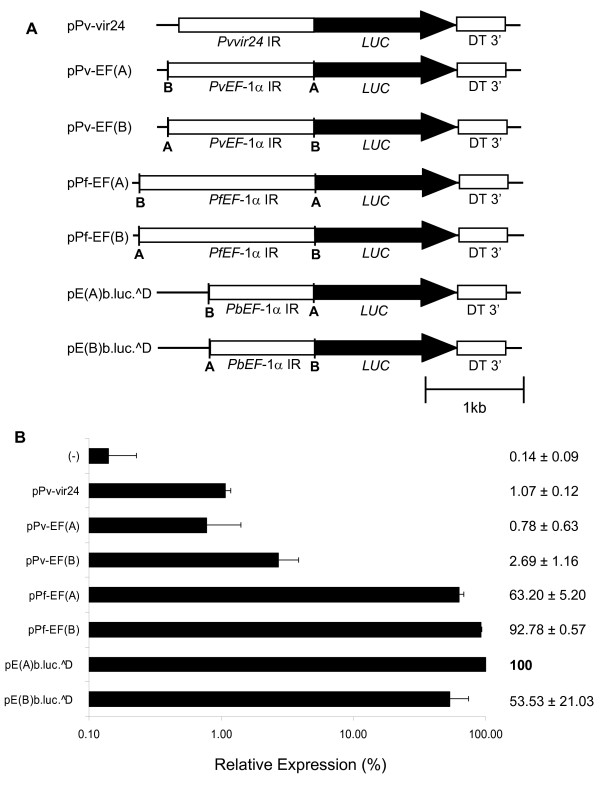
**Entire *P. vivax *intergenic regions are poorly recognized by *P. falciparum***. A. Schematic representation of reporter plasmids with entire intergenic regions. *EF-1α *IR – *elongation factor one alpha *intergenic region; *Pvvir24 *IR – intergenic region between *P. vivax variant genes *23 and 24; *Pv *– *P. vivax*; *Pf *– *P. falciparum*; *Pb *– *P. berghei LUC *– luciferase; DT3' – *P. berghei dihydrofolate reductase *3' UTR. B. Transient transfections in *P. falciparum*. Plasmids were transiently transfected in *P. falciparum *and luciferase activity determined 4 days later. Luciferase expression is represented relative to the activity of pE(A)b.luc.^D. Negative control (-) refers to luminescence measures of the substrate alone or plasmid with luciferase and malaria 3' UTR but no promoter. Log scale was used. Values represent the mean of at least three independent experiments done with two or three different DNA preparations. Bars represent standard deviations.

To guarantee that all *cis *acting regulatory elements within *P. vivax *promoter regions were included in these transient transfection assays, a new plasmid termed pPv-vir24 containing the entire 1.3 kb intergenic region between *vir23 *and *vir24 *genes [34] was constructed (Figure 2A). Transient transfection of pPv-vir24 into *P. falciparum *produced luciferase activity levels significantly above background, suggesting this *P. vivax *intergenic region contains *cis *regulatory elements capable to recruit the *P. falciparum *transcriptional machinery (Figure 2B). However, the reporter activity was about 1% of that observed with the positive control (pE(A)b.luc.^D) indicating poor recognition of these *cis *regulatory elements.

To provide further evidences of this observation, reporter plasmids with the entire intergenic regions of the *ef-1α *genes of *P. falciparum*, *P. berghei *and *P. vivax *were transfected into *P. falciparum*. These genes are orthologs in *Plasmodium *containing two copies each per haploid genome in opposite orientations. In addition, the *ef-1α *intergenic region from *P. berghei *had already been functionally characterized and shown to have promoter activity in both orientations in *P. berghei *and in *P. falciparum *[23,25]. As shown in Figure 2, recombinant plasmids harboring the entire intergenic regions of the *ef-1α *genes from *P. falciparum *and *P. berghei *reported high and comparable luciferase values in either orientation. In contrast, plasmids containing the intergenic region of the *ef-1α *genes of *P. vivax*, cloned in both orientations, reported luc values relative to pE(A)b.luc.^D that were 0.8% (pPv-EF(A)), which is not significantly different from the negative control, and 2.7% (pPv-EF(B)), which is significantly above background (Figure 2B). Together, these results indicate that *cis *regulatory elements within promoter regions of *P. vivax *are poorly or not recognized by the transcriptional machinery of *P. falciparum*.

### A 6 bp motif is divergent in the *P. vivax ef-1α *intergenic region

**Figure 3:**
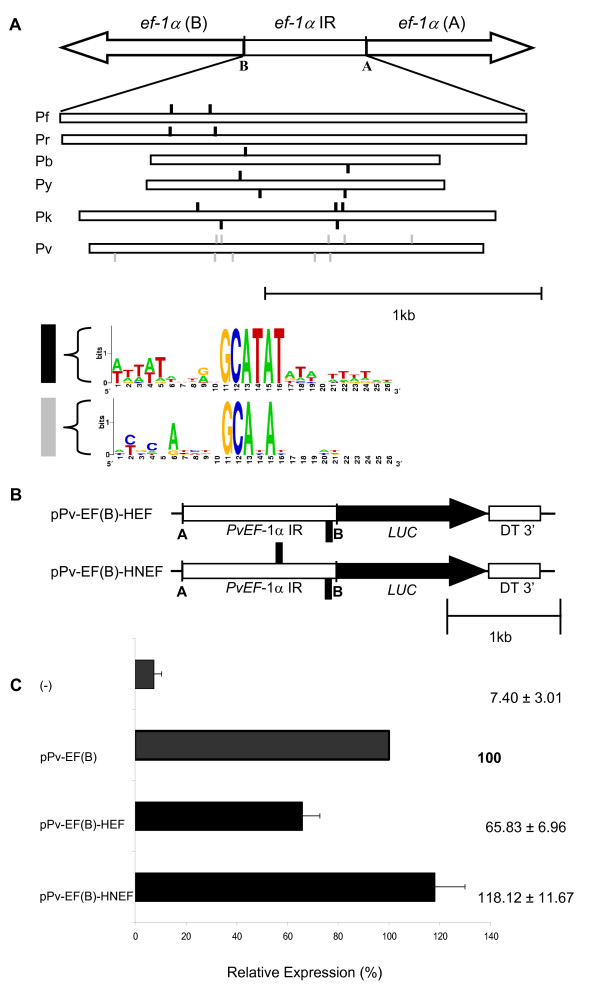
**A conserved motif of the elongation factor one alpha (*ef-1α*) intergenic region can increase the *P. vivax ef-1α *promoter strength**. A. Upper diagram represents the *ef-1α *intergenic region in *Plasmodium*. The Gibbs matrices of the Regulatory Sequence Analysis tools [31] was used to search conserved motifs in the *ef-1α *intergenic region of *P. falciparum *– Pf, *P. reichenowi *– Pr, *P. berghei *– Pb, *P. yoelii *– Py, *P. knowlesi *– Pk and *P. vivax *– Pv. A conserved identical motif – GCATAT – was observed in all species excepting *P. vivax *where positions 4 and 6 are degenerate. Position and orientation of the EF motif along the intergenic regions are shown. B. Reporter plasmids constructed to functionally characterize the motif. The EF motif was cloned into *Hind*III restriction site of pPv-EF(B) to create plasmids pPv-EF(B)-HFE and in the *Nde*I of this one to create pPv-EF(B)-HNFE. Pv *ef-1α *IR – *P. vivax ef-1α *intergenic region; *LUC *– luciferase; DT 3' – *P. berghei dihydrofolate reductase *3' UTR. The EF motif is represented by black squares. C. Transient transfection in *P. falciparum*. Reporter plasmids were transiently transfected in *P. falciparum*. Reporter expression values are represented relative to pPv-EF(B). Negative control (-) refers to luminescence measures of the substrate alone or plasmid with luciferase and malaria 3' UTR but no promoter. Linear scale was used. Values represent the mean of at least three independent experiments done with two or three different DNA preparations. Bars represent standard deviations. Scale is in kilobase-pairs (kb).

An *in silico *approach was undertaken to identify divergent elements in the *P. vivax ef-1α *intergenic region. The *ef-1α *intergenic regions of six *Plasmodium *species (*P. berghei*, *P. falciparum*, *P. knowlesi*, *P. reichenowi*, *P. yoelii *and *P. vivax*) were searched for conserved motifs using the Gibbs sampling and Patser algorithms [30,32]. Some short motifs, conserved in all *Plasmodium *species were found. These included homopolymeric poly(dA)poly(dT) tracts, poly(dAT) tracts and also GC rich motifs (data not shown). Using a matrix length of 27, the motif TGG [G/T]G [C/T]T [A/T] [A/T]GAGGGGTGA [A/G]C [A/C] [G/T]TTAAA was found. This sequence is conserved in the intergenic region of *P. berghei*, *P. falciparum*, *P. reichenowi *and *P. yoelii*, but not in *P. vivax *and *P. knowlesi*, where the central part of the motif is conserved (GAGGGGTG), but the extremities are degenerate. This data indicated that *P. vivax *and its evolutionary related species *P. knowlesi *could share motifs distinct from the species with AT rich genomes. When the matrix length was set to six, a motif (GCATAT) identical among all *Plasmodium *species excepting *P. vivax *(GCANAN) was found. The Patser algorithm was used to determine its position, conservation and copy number in the six *ef-1α *intergenic regions. The motif is present in two to five copies and its position relative to the start codon of the two *ef-1α *genes is maintained in pairs of phylogenetically closely related parasites *P. falciparum*/*P. reichenowi *and *P. berghei*/*P. yoelii*, but not in *P. vivax*/*P. knowlesi *(Figure 3A). This motif, referred to here as the EF motif, has no similarities to known eukaryotic transcription-factor binding sites.

### The EF motif is active in gene expression

**Figure 4:**
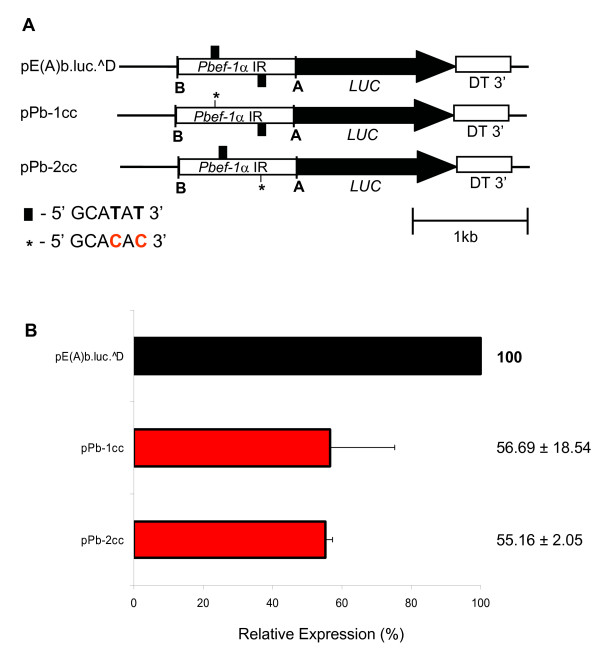
**The EF motif is an active *cis*-acting element of the *P. berghei *ef-1α promoter**. A. Mutations of the EF motifs of *P. berghei ef-1α *promoter. Plasmid pE(A)b.luc.^D had its first or second EF motif mutated (*). B. Transient transfection in *P. falciparum*. Reporter plasmids were transiently transfected in *P. falciparum*. Luciferase activity is expressed relative to pE(A)b.luc.^D. Linear scale was used. Values represent the mean of at least three independent experiments done with two or three different DNA preparations. Bars represent standard deviations.

Since the highly conserved EF motif is degenerate only in *P. vivax*, two complementary approaches were performed to demonstrate that it is active in gene expression. Firstly, two recombinant plasmids containing one or two copies of the EF motif in the *P. vivax ef-1α *intergenic region, were generated (Figure 3B). pPv-EF(B)-HEF, having one copy of the EF motif, significantly reduced the promoter strength to 65% whereas pPv-EF(B)-HNEF, having two copies of the EF motif in different strands, restored reporter activity close to values of the original plasmid pPv-EF(B) (Figure 3C). Secondly, the EF motif in the *P. berghei ef-1α *promoter was mutated to create a sequence similar to the motif of *P. vivax*. To do so, thymidines (T) on positions 4 and 6 of the first or the second copy of the EF motif in plasmid pE(A)b.luc.^D were mutated to cytidines (C) (Figure 4A). Interestingly, luciferase activity significantly dropped to 56% and 55% relative to activity detected with the original plasmid (Figure 4B). Together, this data suggests that both copies of the EF motif are important for promoter activity and that this motif is active in gene expression in *Plasmodium*.

## Discussion

Here, heterologous promoter analysis of the *P. vivax dhfr*, *msp1 *and *vir3 *genes as well as the entire intergenic regions of the *vir23/24 *and *ef-1α *genes in *P. falciparum*, is presented. Noticeable, in spite of being from another human malaria parasite, *cis *regulatory elements within promoter regions of *P. vivax *are poorly or not recognized by the transcriptional machinery of *P. falciparum*. An in silico search of *cis *regularory sequences in *Plasmodium*, identified a conserved identical 6 bp element (GCATAT) in the *ef-1α *intergenic regions of six *Plasmodium *species analyzed. The exception was *P. vivax *where this element was degenerate (GCANAN). Functional analysis of this element through site-directed mutagenesis showed that it is active in gene expression in *Plasmodium*. This data demonstrated that important *cis *regulatory elements are lacking or divergent in the *P. vivax *promoter regions reported here.

Previous studies have shown that *cis *regulatory elements within promoter regions of different species of *Plasmodium *can drive gene expression in heterologous transfection assays [20-23]. Due to the difficulties in maintaining *P. vivax *continuously in culture, heterologous promoter analysis was used to determine if *P. vivax *promoter regions of different sizes and AT-contents are functional in *P. falciparum*. Interestingly the *dhfr, msp*1 and *vir*3 upstream regions analyzed, harboring very distinct AT contents were unable to recruit the transcriptional machinery of *P. falciparum *as determined by the lack of luciferase reporter activity. Size of promoter regions in *Plasmodium *however, varies considerably in genes of the same species and in the same gene among the different species. Thus, it was formally possible that the promoter regions for these *P. vivax *genes reside in longer intergenic regions as those used in the constructs. To exclude this possibility, full intergenic regions of the *P. vivax vir23*/*vir24 *genes and *ef-1α *A/B genes were chosen to determine whether *P. vivax *promoters were poorly recognized by *P. falciparu*m. Luciferase values significantly above the background were detected in plasmids bearing these entire intergenic regions demonstrating that they were able to recruit the transcriptional machinery of *P. falciparum*. Yet, values were about two orders of magnitude lower than plasmids bearing promoter regions from *P. berghei *showing poor recognition. Interestingly, although the *P. falciparum ef-1α *intergenic region is longer and AT richer (1,752 bp/89.7% AT) than the *P. berghei *promoter region (1,052 bp/83.7% AT), reporter activity was not significantly different indicating that *cis *regulatory elements are conserved in these two species. These results suggest that *cis *acting elements are divergent in *P. vivax*. Alternatively, translation efficiency and/or RNA stability of the luciferase transcript in *P. falciparum *is lower in the context of the *P. vivax *5' UTRs.

A bioinformatics approach recently enabled a functional regulatory element in the promoter region of the malaria heat shock protein genes to be identified [19]. A similar approach was used to try to identify divergent motifs in the *ef-1α *promoter region of *P. vivax *that could explain the low luciferase reporter activity in *P falciparum*. The sequence of *ef-1α *of six species of *Plasmodium *was searched for conserved motifs using the Gibbs algorithm. This enabled the identification of a motif, identical in the *ef-1α *intergenic regions of five of these species but degenerate in *P. vivax*. To determine whether this motif, termed EF motif, is active in gene expression, two complementary approaches were taken. First, the conserved sequence of the EF motif was cloned into the *P. vivax ef-1α *promoter region. Unexpectedly, the insertion of one copy decreased luciferase reporter activity to 65% whereas cloning of a second motif restored promoter strength. Second, either EF motif in the *P. berghei ef-1α *promoter of our reference plasmid was mutated as to create an EF motif identical to that of *P. vivax*. Significantly reduced reporter expression was achieved with either motif mutated suggesting that both copies are functional. Regardless, these two approaches suggest that the EF motif is functional in gene expression in *Plasmodium*. Of importance, *in silico *analysis of the *P. vivax *and *P. falciparum *genomes revealed the presence of this motif in several upstream regions further suggesting its functional role in gene expression.

The evolutionary reasons that could explain why *cis *regulatory elements in *P. vivax *promoter regions are poorly or not recognized by *P. falciparum *are presently unknown. The absence of a species barrier for promoters of some species of *Plasmodium *has been demonstrated. Indeed, *dhfr *promoters of *P chabaudi *are functional in *P. falciparum *[20]. Moreover, *P. falciparum *and *P. berghei *promoters are recognized in *P. knowlesi *[21]. Furthermore, a recent study established transient transfection in *P. vivax *using a *P. falciparum *promoter [35]. Thus, promoters from phylogentically distant species are functional in heterologous assays although a direct comparison of promoter strengths in these different systems is lacking. Transfection methodologies for these four *Plasmodium *species have been developed and constructs harbouring promoters characterized for a least three of them are available. It is, therefore, feasible now to pursue functional comparative studies which may identify promoter elements conserved and distinct among parasites of the genus, elucidating many aspects of gene regulation in *Plasmodium*.

## Authors' contributions

MFA carried-on all experiments and drafted the manuscript. HAP conceived and coordinated the study and edited the draft. Both authors read and approved the final version of the manuscript.

## References

[B1] Bozdech Z, Llinas M, Pulliam BL, Wong ED, Zhu J, DeRisi JL (2003). The transcriptome of the intraerythrocytic developmental cycle of *Plasmodium falciparum*. PLoS Biol.

[B2] Coulson RM, Hall N, Ouzounis CA (2004). Comparative genomics of transcriptional control in the human malaria parasite *Plasmodium falciparum*. Genome Res.

[B3] Deitsch KW, Calderwood MS, Wellems TE (2001). Malaria. Cooperative silencing elements in var genes. Nature.

[B4] Gunasekera AM, Patankar S, Schug J, Eisen G, Kissinger J, Roos D, Wirth DF (2004). Widespread distribution of antisense transcripts in the *Plasmodium falciparum *genome. Mol Biochem Parasitol.

[B5] Militello KT, Patel V, Chessler AD, Fisher JK, Kasper JM, Gunasekera A, Wirth DF (2005). RNA polymerase II synthesizes antisense RNA in *Plasmodium falciparum*. RNA.

[B6] Goonewardene R, Daily J, Kaslow D, Sullivan TJ, Duffy P, Carter R, Mendis K, Wirth D (1993). Transfection of the malaria parasite and expression of firefly luciferase. Proc Natl Acad Sci USA.

[B7] Wu Y, Sifri CD, Lei HH, Su XZ, Wellems TE (1995). Transfection of *Plasmodium falciparum *within human red blood cells. Proc Natl Acad Sci USA.

[B8] van Dijk MR, Waters AP, Janse CJ (1995). Stable transfection of malaria parasite blood stages. Science.

[B9] Horrocks P, Dechering K, Lanzer M (1998). Control of gene expression in *Plasmodium falciparum*. Mol Biochem Parasitol.

[B10] Ruvalcaba-Salazar OK, del Carmen Ramirez-Estudillo M, Montiel-Condado D, Recillas-Targa F, Vargas M, Hernandez-Rivas R (2005). Recombinant and native *Plasmodium falciparum *TATA-binding-protein binds to a specific TATA box element in promoter regions. Mol Biochem Parasitol.

[B11] Calderwood MS, Gannoun-Zaki L, Wellems TE, Deitsch KW (2003). *Plasmodium falciparum *var genes are regulated by two regions with separate promoters, one upstream of the coding region and a second within the intron. J Biol Chem.

[B12] Chow CS, Wirth DF (2003). Linker scanning mutagenesis of the *Plasmodium gallinaceum *sexual stage specific gene pgs28 reveals a novel downstream cis-control element. Mol Biochem Parasitol.

[B13] Horrocks P, Lanzer M (1999). Mutational analysis identifies a five base pair cis-acting sequence essential for GBP130 promoter activity in *Plasmodium falciparum*. Mol Biochem Parasitol.

[B14] Dechering KJ, Kaan AM, Mbacham W, Wirth DF, Eling W, Konings RN, Stunnenberg HG (1999). Isolation and functional characterization of two distinct sexual-stage-specific promoters of the human malaria parasite *Plasmodium falciparum*. Mol Cell Biol.

[B15] Mbacham WF, Chow CS, Daily J, Golightly LM, Wirth DF (2001). Deletion analysis of the 5' flanking sequence of the *Plasmodium gallinaceum *sexual stage specific gene pgs28 suggests a bipartite arrangement of cis-control elements. Mol Biochem Parasitol.

[B16] Porter ME (2001). The DNA polymerase delta promoter from *Plasmodium falciparum *contains an unusually long 5' untranslated region and intrinsic DNA curvature. Mol Biochem Parasitol.

[B17] Osta M, Gannoun-Zaki L, Bonnefoy S, Roy C, Vial HJ (2002). A 24 bp cis-acting element essential for the transcriptional activity of *Plasmodium falciparum *CDP-diacylglycerol synthase gene promoter. Mol Biochem Parasitol.

[B18] Voss TS, Kaestli M, Vogel D, Bopp S, Beck HP (2003). Identification of nuclear proteins that interact differentially with *Plasmodium falciparum *var gene promoters. Mol Microbiol.

[B19] Militello KT, Dodge M, Bethke L, Wirth DF (2004). Identification of regulatory elements in the *Plasmodium falciparum *genome. Mol Biochem Parasitol.

[B20] Crabb BS, Cowman AF (1996). Characterization of promoters and stable transfection by homologous and nonhomologous recombination in *Plasmodium falciparum*. Proc Natl Acad Sci USA.

[B21] van der Wel AM, Tomas AM, Kocken CH, Malhotra P, Janse CJ, Waters AP, Thomas AW (1997). Transfection of the primate malaria parasite *Plasmodium knowlesi *using entirely heterologous constructs. J Exp Med.

[B22] Mota MM, Thathy V, Nussenzweig RS, Nussenzweig V (2001). Gene targeting in the rodent malaria parasite *Plasmodium yoelii*. Mol Biochem Parasitol.

[B23] Fernandez-Becerra C, de Azevedo MF, Yamamoto MM, del Portillo HA (2003). *Plasmodium falciparum*: new vector with bi-directional promoter activity to stably express transgenes. Exp Parasitol.

[B24] *Plasmodium vivax *Genome Project. http://www.tigr.org/tdb/e2k1/pva1/.

[B25] de Koning-Ward TF, Speranca MA, Waters AP, Janse CJ (1999). Analysis of stage specificity of promoters in *Plasmodium berghei *using luciferase as a reporter. Mol Biochem Parasitol.

[B26] Trager W, Jensen JB (1976). Human malaria parasites in continuous culture. Science.

[B27] Fidock DA, Wellems TE (1997). Transformation with human dihydrofolate reductase renders malaria parasites insensitive to WR99210 but does not affect the intrinsic activity of proguanil. Proc Natl Acad Sci USA.

[B28] Deitsch K, Driskill C, Wellems T (2001). Transformation of malaria parasites by the spontaneous uptake and expression of DNA from human erythrocytes. Nucleic Acids Res.

[B29] PlasmoDB. http://www.plasmodb.org/plasmo/home.jsp.

[B30] Lawrence CE, Altschul SF, Boguski MS, Liu JS, Neuwald AF, Wootton JC (1993). Detecting subtle sequence signals: a Gibbs sampling strategy for multiple alignment. Science.

[B31] Regulatory Sequence Analysis Tools. http://rsat.ulb.ac.be/rsat/.

[B32] Hertz GZ, Stormo GD (1999). Identifying DNA and protein patterns with statistically significant alignments of multiple sequences. Bioinformatics.

[B33] WebLogo. http://weblogo.berkeley.edu/logo.cgi.

[B34] del Portillo HA, Fernandez-Becerra C, Bowman S, Oliver K, Preuss M, Sanchez CP, Schneider NK, Villalobos JM, Rajandream MA, Harris D (2001). A superfamily of variant genes encoded in the subtelomeric region of *Plasmodium vivax*. Nature.

[B35] Pfahler JM, Galinski MR, Barnwell JW, Lanzer M (2006). Transient transfection of *Plasmodium vivax *blood stage parasites. Mol Biochem Parasitol.

